# Biomimetic pHEMA Hydrogels as an Alternative Cartilage-like Model Material for Biotribological Evaluations

**DOI:** 10.1021/acsomega.5c05569

**Published:** 2025-09-15

**Authors:** Zuzana Kadlecova, Ivana Chamradova, Klara Tuslova, David Rebenda, Pavel Cipek, Jan Gregora, Alexandra Stredanska, Yoshinori Sawae, Premysl Mencik, Martin Vrbka, Lucy Vojtova

**Affiliations:** † Advanced Biomaterials Group, Central European Institute of Technology, 48274 Brno University of Technology, 621 00 Brno, Czech Republic; ‡ Biotribology Research Group, Faculty of Mechanical Engineering, Brno University of Technology, 616 69 Brno, Czech Republic; § Centre of Polymer Systems, University Institute, Tomas Bata University in Zlin, 760 01 Zlin, Czech Republic; ∥ Department of Mechanical Engineering, Faculty of Engineering, Kyushu University, 744 Motooka, Nishi-ku, 819-0395 Fukuoka, Japan; ⊥ Institute of Materials Chemistry, Faculty of Chemistry, Brno University of Technology, 612 00 Brno, Czech Republic

## Abstract

Poly­(vinyl alcohol) (PVA) has been widely explored as a model material for articular cartilage (AC) in biotribological evaluations. However, PVA hydrogels prepared by freeze–thawing or cast-drying methods have limitations in precisely controlling their elasticity parameters and may require reinforcement to enhance their mechanical performance and change their transparency, required in some tribological measurement setups by using fluorescence methods. To overcome these issues, poly­(hydroxyethyl methacrylate) (pHEMA) hydrogels have been introduced as alternatives. In our study, pHEMA hydrogels synthesized using free-radical polymerization with blue light under two different atmospheres (nitrogen N_2_ and air) were compared with natural samples of articular bovine cartilage. The optical, mechanical, swelling, and tribological properties demonstrate the superior properties of pHEMA, which may result in the replacement of the currently used PVA-based model in future studies. Synthesis under a nitrogen atmosphere (*pHEMA N*_2_) resulted in the formation of smooth-surfaced hydrogels, whereas synthesis under a laboratory atmosphere (*pHEMA air*) resulted in the formation of wrinkled-surfaced hydrogels. The swelling of both the hydrogels and AC followed first-order kinetics. Pin-on-plate biotribology measurements showed that the coefficient of friction of the wrinkled-surface hydrogels resembled that of AC. Our results showed that pHEMA-based hydrogels are suitable biotribological AC models for a better understanding of the biological functions of bovine AC. This knowledge brings new insights into cartilage complex mechanisms and might be applied in both biomedical and engineering applications.

## Graphical abstract

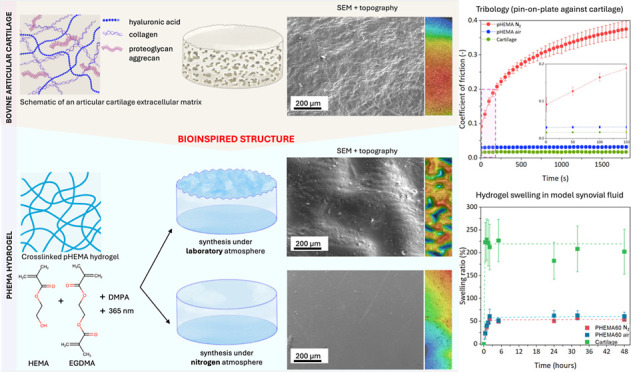

## Introduction

Human articular cartilage (AC) has a vital function in absorbing joint pressure during movement and minimizing friction between bones.^1^ AC is a complex form of connective tissue primarily composed of hyaline cartilage, which plays a vital role in bearing weight, absorbing shock, and facilitating lubrication in joints throughout the body to minimize friction.^2^ AC is a specialized type of hyaline cartilage, the most common cartilage in the human body, usually measuring 2–4 mm in thickness.^3^ It consists of approximately 60–80% water, 15–22% collagen, 5–10% chondrocytes, 4–7% proteoglycans, minerals (less than 4%), and matrix proteins (less than 1%),^4,5^ forming a complex composition that forms a vascular-free structure without nerves or lymphatics.^6^ AC is a porous, viscoelastic substance comprising three main phases: a solid extracellular matrix (ECM) phase, a fluid phase containing water (interstitial fluid), and an ion phase (composed of dissolved electrolytes with both negative and positive charges, less than 1%). These phases allow the tissue to withstand compressive forces.^2,7^ As it is exposed to different types of external forces, such as compression, tension, shear, and friction, it exhibits impressive strength (ranging from 9 to 40 MPa) and toughness (with a fracture energy of 1000 to 15,000 J·m^–2^) and shows a significant strain at failure (ranging from 60 to 120%).^8–10^ AC is an anisotropic material with four specific zones: the superficial (or tangential) zone (representing 10–20% of the AC thickness), middle (or transitional) zone (accounting for 40–60% of the total cartilage volume), deep (or radial) zone (constituting approximately 30% of the AC volume), and calcified zone.^8^ Various structural elements within cartilage, such as collagen fibers, proteoglycans, and water content, dictate its biophysical characteristics and ability to endure mechanical stresses. The integrity of the cartilage is influenced by mechanical loading, and research has highlighted the necessity of replicating the biomechanical environment of chondrocytes for effective tissue engineering and repair.^11^ Additionally, the sluggish metabolic rate of cartilage, a consequence of its lack of blood supply, hampers its regenerative capacity, thus contributing to the onset of chronic conditions such as osteoarthritis (OA).^12^ Tissue engineering offers a promising avenue for investigating the mechanisms of cartilage regeneration by leveraging cells and hydrogels to promote chondrogenic differentiation in laboratory settings, thereby enhancing their therapeutic potential for in vivo applications.^1,13,14^

To understand the tribology of cartilage and lubrication mechanisms for future implications and possible applications of artificial cartilage in clinical practice, several materials have been investigated as model materials for tribological measurements and research. Hydrogels based on poly­(vinyl alcohol) (PVA) and its copolymers have garnered interest in various scientific investigations.^15^ The use of PVA hydrogels presents several advantages, such as favorable biocompatibility, reduced friction, and capacity to simulate the behavior of natural tissues.^14,16,17^ Typically, the elasticity parameters of PVA hydrogels produced through conventional freeze–thaw (FT) or cast-drying (CD) techniques cannot be precisely regulated because they are influenced mainly by two factors: molecular weight and the number of freezing cycles.^18,19^ However, the concentration of PVA in water also plays a crucial role in determining the mechanical properties of the resulting hydrogel. Increasing the PVA concentration generally enhances crystallinity and mechanical strength due to improved polymer chain orientation and intermolecular interactions. Optimal enhancement is usually observed within a concentration range of 10–20% w/v, depending on the molecular weight and degree of hydrolysis. Beyond this threshold (typically above 20–25%), further increases in concentration can lead to reduced chain mobility, increased structural heterogeneity, and internal stress, ultimately resulting in decreased crystallinity and mechanical integrity, such as brittleness and lower flexibility.^20^

Nevertheless, the primary drawback is the subpar mechanical characteristics of these hydrogels, which impede their potential to effectively substitute natural cartilage.^21–23^ These include low tensile strength (typically below 20 MPa), insufficient wear resistance, and a high coefficient of friction (COF)—all critical factors for load bearing and frictional applications.^24,25^ To counter this constraint, researchers have developed diverse methodologies to improve the mechanical properties of PVA hydrogels, including the integration of reinforcing fibers, such as poly­(*p*-phenylene-2,6-benzobisoxazole) (PBO) nanofibers,^22^ phosphate glass fibers (PGF), poly­(ethylene glycol) (PEG), ramie fibers,^26^ cellulose,^27^ poly­(ethylene) (PE),^28,29^ and biodegradable glass fibers.^14^ Numerous studies have introduced bionic and hybrid hydrogels by combining PVA with polymers, such as poly­(acrylamide) (PAM),^30^ poly­(acrylic acid) (PAA),^17^ sodium phytate (PANa),^31^ gum Karaya,^32,33^ and poly­(ethylene glycol)/glycerol^34,35^ to increase their efficacy and drug-conveying capacities.^18,30^ In essence, PVA-based hydrogels and their blends continue to advance, providing reduced friction, elevated mechanical robustness, and improved drug delivery capabilities, notwithstanding the pivotal impact of preparation methodologies and the need for further optimization which remain notable challenges.^36^ In particular, the development of PVA copolymers to enhance mechanical properties poses several difficulties, including copolymer compatibility, preservation of biocompatibility, and the complexity of achieving uniform cross-linking, as more intricate systems may lead to gel heterogeneity or incomplete network formation. Additionally, ensuring reproducibility and maintaining favorable rheological properties, processability, and long-term stability, especially in terms of swelling and degradation, represent further critical aspects that must be addressed.^25,37,38^

Frictional measurements in pin-on-plate^9,39^ or pin-on-disc^9^ configurations are commonly used to study the lubrication mechanism of AC. These methods assess the evolution of the COF between two AC samples,^1^ sometimes replaced by a glass^40^ or PVA hydrogel.^41^ However, these studies do not provide sufficient insight into the underlying mechanisms necessary to understand the lubrication regimes of AC. Our research group has developed a methodology^42^ that combines frictional measurements in a pin-on-plate configuration with simultaneous in situ observation of the contact area by fluorescence microscopy. This method enables the direct observation of fluorescently labeled proteins and HA clusters in the AC contact and the formation of a boundary lubricating layer on the AC surface. This methodology requires one of the contact surfaces to be transparent. The optical glass used thus far is not an ideal substitute in terms of mechanical properties, porosity, and so on. One of the most widely accepted theories of AC lubrication–hydration lubrication^43^ assumes the interaction between phospholipids in synovial fluid (SF) and HA attached to the AC surface. This interaction is crucial for exposing the positively charged phospholipid heads and for the diffusion-driven exchange of water molecules in their hydration shells, leading to the extremely low COF values, around 0.010, reported for AC.^44^ However, HA or phospholipids cannot bind to inert glass, making cartilage-on-glass an inadequate substitute for analyzing AC hydration lubrication mechanisms.

Our study aims to develop a material that can be used as a substitute for the currently used PVA hydrogel or optical glass in tribological testing with a pin-on-plate tribometer and in our setup using fluorescence microscopy. For the material to be suitable for superlubricity measurements, in combination with observations using fluorescence microscopy, it must be transparent, with well-defined mechanical, chemical, and swelling properties that are ideally similar to those of AC.

The poly­(hydroxyethyl methacrylate) (pHEMA)-based hydrogels have been widely used in various biomedical application, since their development in the 1960 for the soft hydrogel contact lenses application by Wichterle and Lím.^45^ They exhibit excellent flexibility and transparency as well as excellent mechanical strength and biocompatibility. The primary benefit of pHEMA-based hydrogels comes from their easily customizable mechanical and optical properties by adjusting a few key parameters, such as the cross-linking mechanism or monomer concentration. This allows for the delivery of the desired mechanical properties, including elasticity, rigidity, stability, and appearance.^46^ With the ability to make materials either transparent or translucent, pHEMA-based hydrogels offer a versatile solution for a wide range of tribological applications. In general, pHEMA hydrogels can be produced by radical polymerization with adjustable swelling and mechanical properties, providing good reproducibility and reduced cost compared with natural hydrogels.^47^ Therefore, they are ideal for replacing the currently prevailing model PVA hydrogels, mainly because of their low friction coefficients and mechanical properties, which resemble those of the natural cartilage.^48^ Nevertheless, pHEMA materials may also require reinforcement for improved mechanical performance, similar to that of PVA hydrogels reinforced with PBO nanofibers.^22^ However, enhancements in pore size, stability, and rigidity are essential for enhancing cell conductivity.^49^ Additionally, pHEMA can be altered to facilitate drug delivery to the deep zones of cartilage, thus serving as an efficient carrier for treating osteoarthritis.^49,50^

## Materials and Methods

### Materials

To prepare pHEMA hydrogels, 2-hydroxyethyl methacrylate (HEMA) was purchased from Sigma-Aldrich (Germany), 2,2-dimethoxy-2-phenylacetophenone (DMPA) from Acros Organics (Thermo Fisher Scientific, UK), and ethylene glycol dimethacrylate (EGDMA) from Sigma-Aldrich (Germany). The Milli-Q water Type 1 (ISO 3696) was prepared using a Millipore purification system (Milli-Q Academic, France).

The physiological solution (PS) was purchased from B. Braun (Germany), and hyaluronic acid HySilk (HA) with a molecular weight of 820–1020 kDa for the preparation of SF was purchased from Contipro a.s. (Czech Republic). l-α-Phosphatidylcholine was purchased from Sigma-Aldrich (USA). Potassium chloride, sodium chloride, potassium dihydrogen phosphate, and disodium hydrogen phosphate dodecahydrate were purchased from LachNer (Czech Republic) for the preparation of phosphate buffer (phosphate buffered saline (PBS)). Bovine serum albumin and γ-globulin were purchased from Sigma-Aldrich (USA). A high-intensity UV lamp (T8, 18 W, 60 cm) with a wavelength of 365 nm was purchased from Eurolite (Germany).

### Methods

#### Synthesis of HEMA Hydrogel Samples

Transparent pHEMA hydrogels were prepared by free-radical polymerization of monomer HEMA, cross-linker EGDMA, photoinitiator DMPA, and Milli-Q water under an inert (nitrogen) atmosphere or in air at laboratory temperature. The synthesis of water-based pHEMA hydrogels was inspired by our previous work,^51^ in which monomer HEMA was added at 60% w/w, DMPA was adjusted to 0.25–0.5% w/w, and cross-linker 0.25–1% w/w. First, the photoinitiator, monomer, water, and the cross-linker were mixed. The liquid mixture was transferred to a mold with a fixed volume attached to tribological glass at the bottom, where polymerization occurred. The solution was irradiated with a 365 nm, 15 W UV lamp (emission in the visible region) at a fixed distance of 10 cm from the center of the samples, with an irradiation period of less than 20 min. The synthesis was performed either under a nitrogen atmosphere in a plastic container with a closed lid under constant nitrogen flow (*pHEMA N*_2_) or under laboratory atmosphere, 23 °C, humidity 28% (*pHEMA air*).

The freeze–thaw poly­(vinyl alcohol) (FT PVA) hydrogels were prepared according to our previous publication^52^ without further modification.

#### Preparation of Cartilage Samples

Cartilage samples were obtained from bovine femoral heads within 24 h of slaughter in a local butcher shop. The fresh joints were stored in a refrigerator at 4 °C prior to sample extraction. For the static swelling tests, cartilage specimens were obtained from the center of the femoral head using a precise cutting tool (standardized punch, diameter 4 mm)^53^ to create uniform cartilage disks without subchondral bone. For tribological measurements, the femoral head was cut with an oscillating saw to harvest plate samples (approximately 45 × 20 mm in size). Cartilage pins with a diameter of approximately 9.7 mm were pressed from some of these plates by using a custom-designed punch. Depending on the condition and size of the femoral head, a maximum of one cartilage plate or two pin samples were obtained from each femoral head. The cartilage plates and pins were removed, along with the subchondral bone, to clamp the tribometer. The samples were stored in PBS at −22 °C before the experiments to prevent cartilage degradation.

#### Tensile Testing

To obtain reference values for the tensile stress (σ_t_), tensile strain (ε_0_), and Young’s modulus (*E*_t_) of the samples, a series of uniaxial tensile tests following the ISO 527 standard were conducted using a ZwickRoell Z010 material testing machine. A 500 N load cell was used to test the pHEMA hydrogel samples in a dogbone 5A shape. The tests were performed at a preload speed of 5 mm·min^–1^ to reach a preload force of 1 N, followed by a load speed of 2 mm·min^–1^ in the linear region of tensile testing, and finally, 20 mm·min^–1^ to achieve a 50% decrease in the maximal force (*F*_max_). The preload was applied to align the hydrogel samples within the Zwick testing apparatus. Upon reaching the predefined preload value, the actual measurement process commenced. This procedure considers the preload value as the baseline (zero point) for stress. Correspondingly, the strain is defined as zero at this stage, initiating the measurement interval for the elastic modulus in the normative range of 0.05–0.25% strain. Young’s modulus (*E*_t_) was calculated from the slope of the linear-elastic region of the stress–strain curve using eq 1

1 $$E_{t}=\frac{\sigma_{1}}{\varepsilon_{2}-\varepsilon_{1}}$$

where ε_1_ and ε_2_ are values of stress (MPa) corresponding to relative deformations between 0.0005 and 0.0025 (−).

The stress was calculated using eq 2

2 $$\sigma_{i}=\frac{F_{i}}{A}$$

where *F*_*i*_ is the applied force at the given time point and *A* is a cross-section area of the “neck” part of the dog-bone sample.

The deformation was calculated using eq 3

3 $$\varepsilon_{i}=\frac{L_{i}-L_{0}}{L_{0}}$$

where *L*_*i*_ is the actual sample elongation (mm) and *L*_0_ is the initial length of the measured part of the sample (mm).

According to the ISO 527 standard, the initial length *L*_0_ was set to 50 mm. The experiments were conducted in a series of *n = 5* tests at laboratory temperature (23 °C).

#### Attenuated Total Reflectance Fourier-Transform Infrared Spectroscopy (ATR-FTIR)

Fourier-transform infrared spectroscopy was used to characterize the chemical composition of the dried pHEMA sample surface. ATR-FTIR Hyperion 3000/Vertex70v (Bruker, USA) with a germanium ATR crystal was used, and the measurements were performed under evacuated conditions. Spectra in the wavenumber range of 4000–650 cm^–1^ were obtained by averaging 100 scans per sample with a resolution of 4 cm^–1^. The spectra were normalized using min–max normalization (OPUS software, Bruker, USA).

#### Static Swelling Test

PHEMA hydrogels and cartilage samples in the form of 4 mm discs were left to dry overnight in a laboratory oven (Ecocell 111, Thermo Fisher Scientific, Czech Republic) at a temperature of 50 °C until they reached a constant weight. The average thickness of pHEMA hydrogels was 2.0 ± 0.2 mm, and the thickness of the cartilage samples was 2.2 ± 0.2 mm. A solution of SF comprising 0.5% w/v HA in Milli-Q water was utilized, given the extended testing period during which γ-globulin and bovine serum albumin may deteriorate. The samples were then immersed in excess physiological water or SF and incubated at 37 °C. Subsequently, the samples were wiped with a lint-free cloth, and the amount of water absorbed was determined using gravimetric analysis at various time intervals, namely, 30, 60, and 90 min, as well as at 2, 5, 24, and 48 h. The experiments were performed in a series of *n* = 5 tests, and the swelling of the hydrogels was calculated using eq 4

4 $$Swelling\;ratio\;\left(\%\right)=\frac{W_{1}-W_{0}}{W_{0}}\times 100$$

where *W*_0_ is the initial weight of the dried sample and *W*_1_ is the weight of the swollen sample at the defined time interval.

A first-order swelling model was used to fit the experimental data and to examine the swelling control process. Equation 5 was used to calculate first-order kinetics.^54,55^

5 $$\frac{dS}{dt}=k_{1}\cdot \left(S_{\max}-S\right)$$

where *k*_1_ is the rate constant for first-order swelling kinetics, *S* is the degree of swelling at a specific time point, and *S*_max_ is the degree of swelling at equilibrium.

The first-order (*k*_1_) swelling constant and *S*_max_ were calculated by fitting the experimental data to the model in Excel using the function “Solver” with the parameters set to be larger than or equal to zero.

#### Morphology

The surface morphologies of dried PHEMA hydrogel and cartilage samples were examined by using scanning electron microscopy (SEM) and noncontact optical profilometry. To conduct the SEM experiments, the samples were dried overnight in a laboratory oven Ecocell 111 at 50 °C until a constant weight was obtained. The surfaces of the HEMA and cartilage samples were coated with a 15 nm-thick gold layer using a Leica EM ACE600 coater (Leica, Germany). Imaging was performed using a MIRA3-XMU microscope (Tescan, Czech Republic) at suitable magnifications and a working distance at a high voltage of 5 kV. Images were processed by using ImageJ2 software (National Institutes of Health, USA). Surface roughness measurements were made using a Keyence VHX-7000 3D optical scanning microscope (Keyence, Belgium). For each sample, a surface area with a size of approximately 1 × 5 mm was scanned by performing a series of overlapping horizontal scans at 300× magnification. The area surface parameters—arithmetical mean height (*S*_a_) and maximum height (*S*_*z*_)—were evaluated from the entire scanned area. The profile filter cutoff wavelength was set to λ_c_ = 2.5 mm.

#### Tribology

To determine the frictional properties of pHEMA hydrogels, reciprocating sliding tests were conducted using a universal tribometer (UMT Tribolab, Bruker, USA). Sliding tests employed a pin-on-plate configuration shown in Figure 1 to examine the COF as a function of time.^56^ The contact pair consists of a stationary plate composed of a pHEMA hydrogel and a moving cartilage pin. For reference, a sliding test was performed with a cartilage pin sliding against a plate. During the test, the cartilage pin was subjected to a constant load of 5 N and a reciprocating sliding motion at a speed of 10 mm·s^–1^ with a stroke length of 12 mm. Each test lasted 30 min. The contact pair was fully flooded with artificial SF and the lubricant with the plate sample was heated to 37 °C.^57^ The experiments were performed in *n* = 5 tests per sample type.

**Figure 1:**
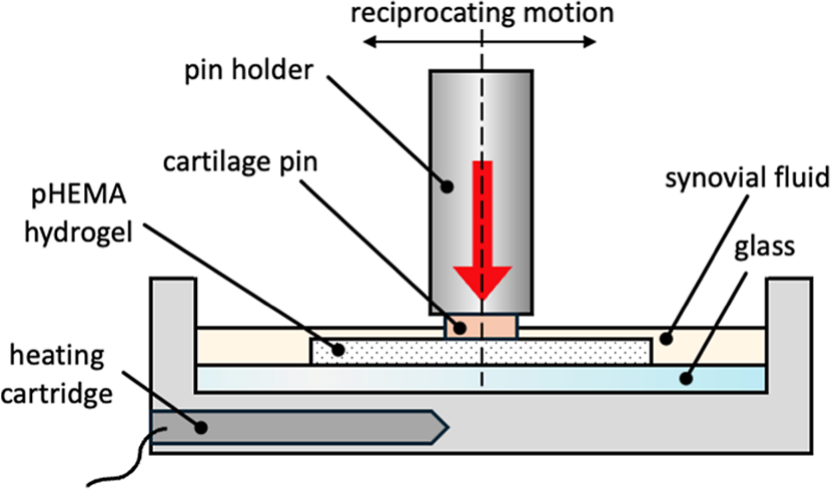
Scheme of a pin-on-plate setup for the determination of COF of the pHEMA hydrogels against a cartilage pin.

For tribological testing, artificial SF was prepared according to the method described by Galandáková et al. (2017).^58^ Briefly, 2.5 mg·mL^–1^ HA, 0.15 mg·mL^–1^ l-α-phosphatidylcholine, 20 mg·mL^–1^ bovine serum albumin, and 3.6 mg·mL^–1^ γ-globulin were added to a PBS solution (2.68 mM potassium chloride, 136.89 mM sodium chloride, 0.56 mM potassium dihydrogen phosphate, 23.59 mM disodium hydrogen phosphate dodecahydrate) and mixed until dissolved.

#### Estimation of Permeability by Stress Relaxation Tests

The permeability of pHEMA was estimated through stress-relaxation tests conducted using an Autograph AGS-X testing machine (Shimazu, Japan) equipped with a 100 N capacity SM-100n-168 load cell. The pHEMA specimens, with dimensions of 20 × 10 mm and thicknesses of 2.50 mm (for *pHEMA air*, *n* = 3) and 2.66 mm (for *pHEMA N*_2_, *n* = 3), were subjected to compression under a 30 mm diameter aluminum cylinder. The applied strain was maintained at 15% and the relaxation time was set to 21,600 s. Throughout the compression tests, the samples were submerged in a Milli-Q water bath to replicate physiological conditions. The permeability was estimated using the method described by Yamaguchi et al. (2018)^59^ based on the relaxation function *E*(*t*) in eq 6.

6 $$E\left(t\right)=E_{\infty }+E_{1}\cdot e^{t/\tau_{1}}+E_{2}\cdot e^{t/\tau_{2}}$$

The parameters were determined using the least-squares method with the maximum load time designated as *t* = 0. The shear storage modulus *G* was calculated using eqs 7 and 8.

7 $$G=\frac{\sigma_{z}\left(t=0\right)}{3\varepsilon_{z}}=\frac{E_{\infty }+E_{1}+E_{2}}{3}$$

$$\sigma_{z}\left(t=0\right)=E_{\infty }\varepsilon_{z}=\frac{3\cdot G\cdot K}{K+\frac{G}{3}}\cdot \varepsilon_{z}$$ 8

where *E*_∞_ is the relaxed modulus, ε_*z*_ is applied strain, *G* is the shear storage modulus, *K* is the osmotic modulus.

The osmotic modulus *K* was calculated using eq 9.

9 $$K=\frac{G\cdot E_{\infty }}{9\cdot G-3{\cdot E}_{\infty }}$$

where *G* is the shear storage modulus and *E*_∞_ is the relaxed modulus.

The relationship between the relaxation time τ_2_ and diffusion coefficient (DC) is expressed as in eq 10.

10 $$\tau_{2}=\frac{1}{DC}\cdot \frac{1}{\pi^{2}\left(\frac{1}{L^{2}+W^{2}}\right)}$$

where *L* and *W* are the length and width of the sample, respectively.

The values were calculated by fitting the experimental data to the model in Excel using the function “Solver”. Finally, the coefficient of permeability, *k*, was calculated by using eq 11.

11 $$k=\frac{DC}{\left(1-\varphi \right)\cdot \left(K+\frac{4}{3\cdot G}\right)}$$

#### Statistical Analysis

Data are presented as the mean ± standard deviation (SD) unless otherwise specified. The unpaired Student’s *t*-test was used to compare two groups, whereas one-way analysis of variance followed by Tukey’s multiple comparison test was used to analyze more than two groups. The level of statistical significance was set at *p* < 0.05. The data were assessed using the OriginPro 2020b software (OriginLab Corporation, USA).

## Results and Discussion

### Appearance and Morphology

Polymerization parameters, including solvents, additives, and conditions, significantly affect the morphological characteristics of pHEMA hydrogels, indirectly leading to differences in the mechanical strength or swelling capacity, which are critical factors in their suitability for biomedical applications.

First, the concentrations of the HEMA monomer and DMPA initiator were optimized to ensure the good optical properties of the hydrogels. As shown in Figure 2, the pHEMA hydrogels had a homogeneous structure, which contributed to their good transparency^60^ at the selected concentrations of HEMA and DMPA upon preparation. However, upon submersion into Milli-Q water, most of the prepared pHEMA hydrogels became opaque with a milky white appearance. Therefore, based on stiffness and transparency perceived through visual observation, pHEMA hydrogels with 60% w/w HEMA monomer, 0.5% w/w DMPA initiator, and 1.0% w/w EGDMA cross-linker content were used in this study.

**Figure 2:**
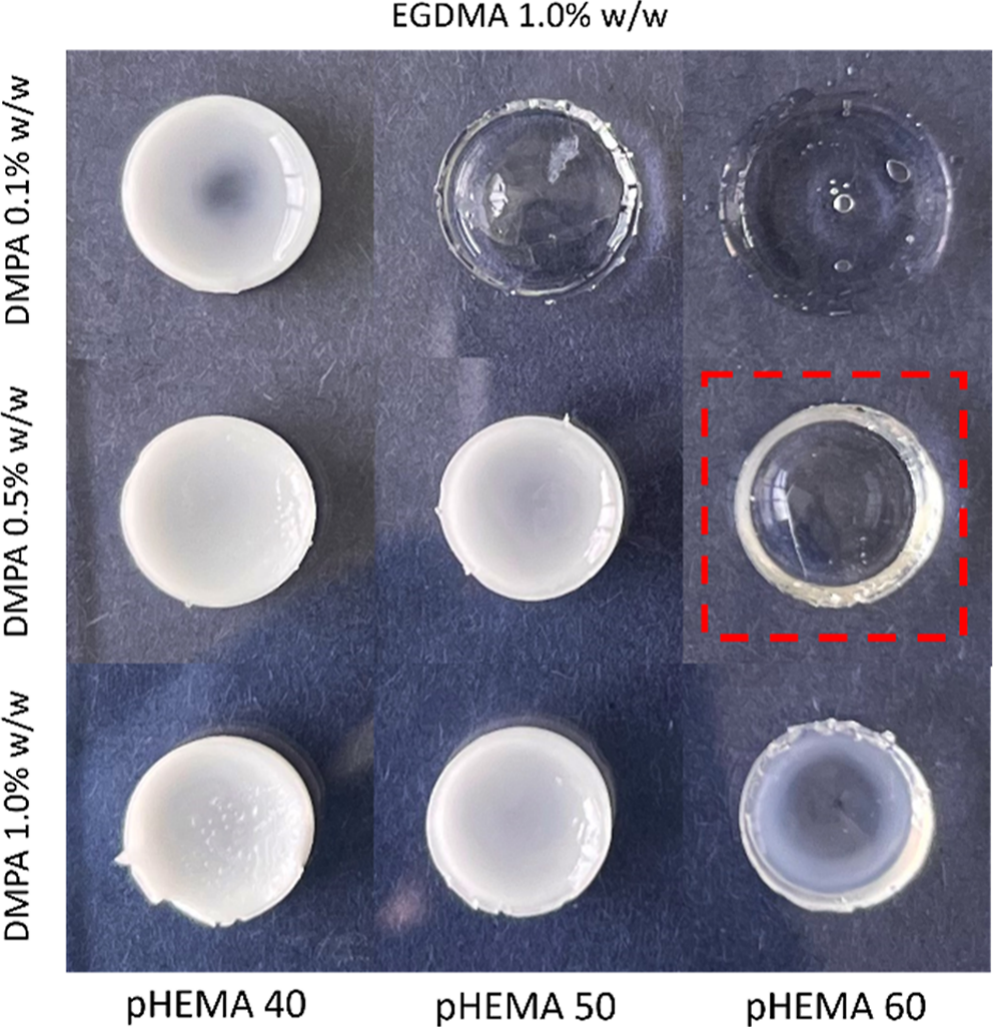
Hydrogels based on different HEMA monomer concentrations (% w/w) and initiator DMPA concentrations (% w/w).

Furthermore, we investigated the influence of environmental conditions on the surface morphology of the pHEMA hydrogels. Upon casual inspection, morphological disparities were observed between the samples based on the selected formulation prepared in a laboratory setting (*pHEMA air*) and those prepared under a nitrogen atmosphere (*pHEMA N*_2_). The morphologies of the samples are shown in Figure 3.

**Figure 3:**
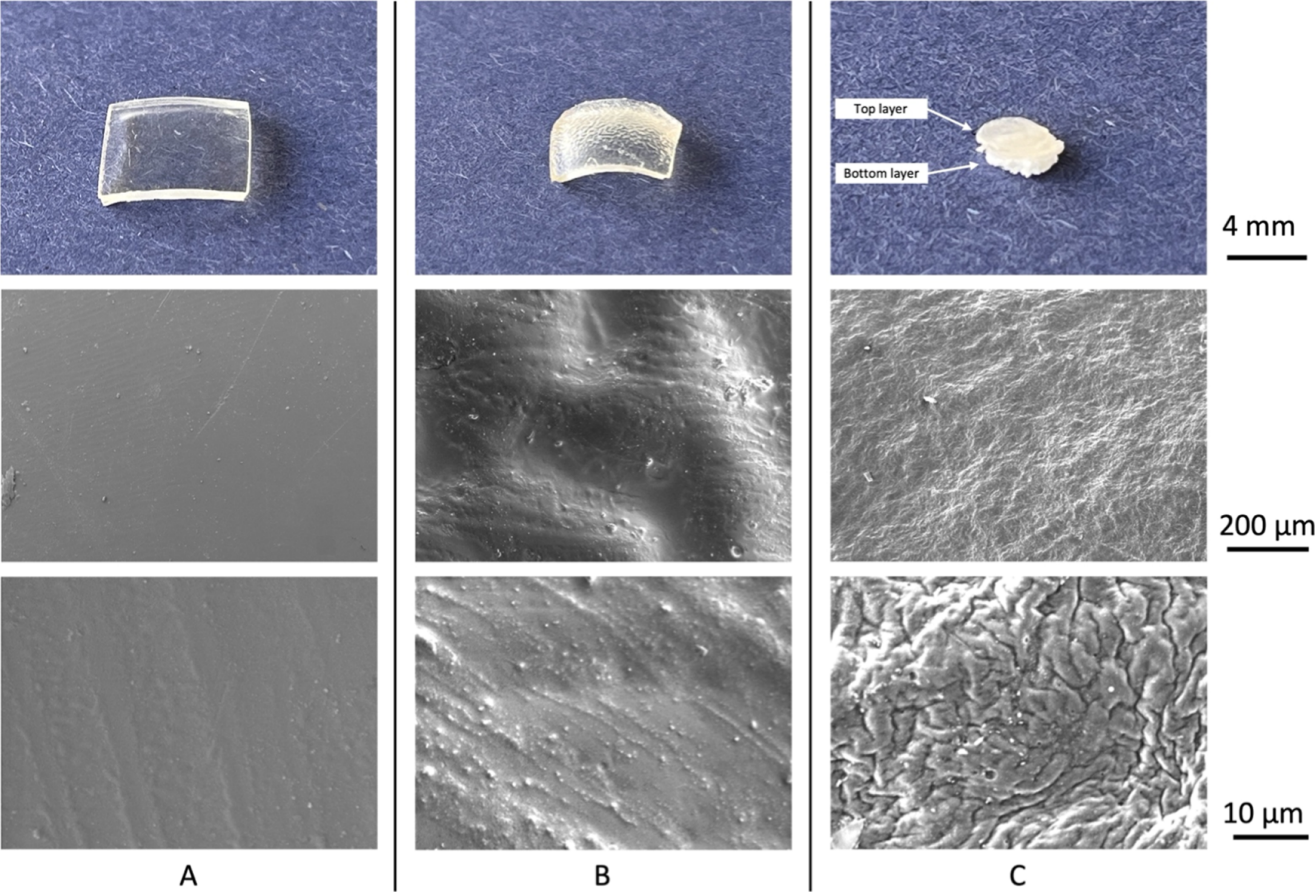
Images of the dried samples: (A) *pHEMA N*_2_, (B) *pHEMA air*, and (C) bovine cartilage of the original size (first row) and SEM of their according surfaces with 200 and 10 μm scale bars (second and third row, respectively).

Observing the samples with SEM, the *pHEMA air* and cartilage samples exhibited a wrinkled surface, whereas the *pHEMA N*_2_ samples had smooth surfaces, as observed by Podestà et al. (2005).^61^ Similar wrinkling patterns were generated on pHEMA by Yun et al. (2019)^62^ under laboratory conditions. Our results show that wrinkling can be controlled not only by the initiator concentration and layer thickness in the study by Yun et al. but also by changes in atmospheric conditions.

Examination of the composition of bovine AC revealed that the surface displayed a more pronounced level of wrinkling than both *pHEMA air* and *pHEMA N*_2_. However, the AC and pHEMA hydrogels exhibit disparate structural characteristics. As shown in Figure 3C, the top layer of the AC is translucent, whereas the deeper zones of the cartilage are spongy. Despite this, *pHEMA N*_2_ exhibited properties considerably closer to those of natural AC than those of *pHEMA air*.

The wrinkled surfaces of *pHEMA air* and AC and the smooth surface of *pHEMA N*_2_ were further investigated by surface roughness measurements. Figure 4A shows color scale images of surface textures, while Figure 4B shows results of the arithmetical mean height *S*_a_ and maximum height *S*_*z*_. The surface roughness of wrinkled *pHEMA air* was substantially higher than that of *pHEMA N*_2_ and AC with measured values of *S*_a_ = 8.96 μm and *S*_*z*_ = 106.31 μm. *pHEMA N*_2_ showed results much closer to those of AC. Reported values of *S*_a_ = 1.17 μm and *S*_*z*_ = 20.82 μm were measured for *pHEMA N*_2_ and *S*_a_ = 2.47 μm and *S*_*z*_ = 52.23 μm for AC.

**Figure 4:**
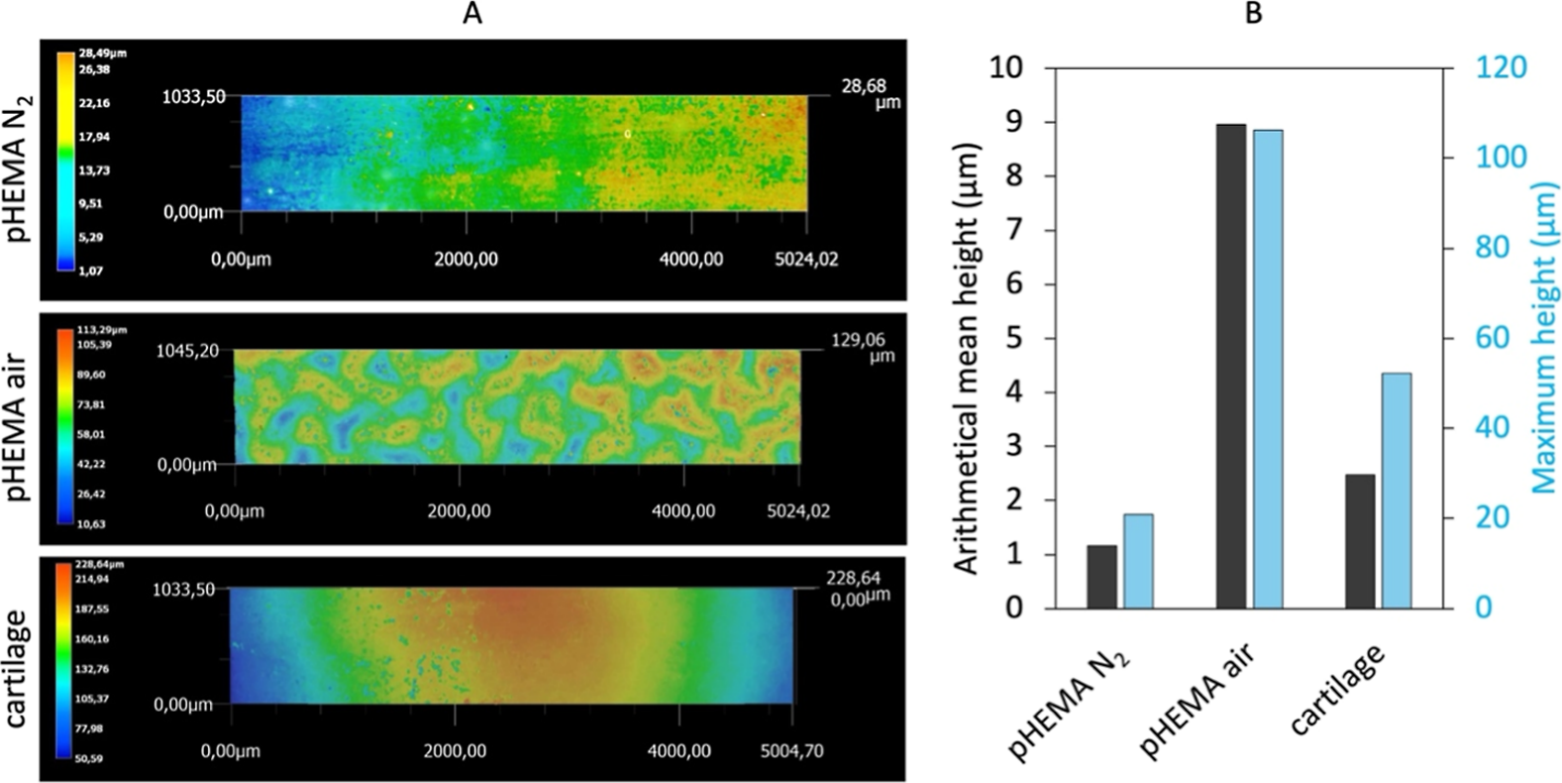
Surface roughness measurements: (A) images of scanned surface with height deviations, (B) arithmetical mean height and maximum height for pHEMA samples and AC.

In comparison with the literature, different methods have been employed to measure AC roughness, yielding different results depending on the technique used. Our results are in good agreement with the study by Smyth et al.,^63^ who reported an average roughness value *R*_a_ between 1.6 and 2.29 μm measured by stylus profilometer. On the other hand, Ghosh et al.^64^ reported surface roughness *S*_a_ in the range of 183–261 nm measured by SEM, and between 86 and 136 nm measured by atomic force microscopy. The surface roughness of pHEMA hydrogels is known to change significantly depending on the hydration state.^65^ Therefore, samples were immersed in water, and the surface was dried with a hand towel prior to measurement in order to avoid reflections. Typical transparent pHEMA hydrogels have surface roughness parameters in the order of units to tens of nanometres^66,67^ due to their application in contact lenses and artificial corneas. Nevertheless, our goal was to substitute AC in the tribological measurements. The role of AC roughness in cartilage-on-cartilage lubrication is of paramount importance, leaving *pHEMA N*_2_ more closely mimicking AC surface roughness than *pHEMA air*.

### Structural Characterization

Figure 5A shows the ATR-FTIR spectra of the pHEMA precursor, HEMA monomer, DMPA photoinitiator, and EGDMA cross-linker. The monomer HEMA has characteristic transmission wavenumbers at 1637 and 816 cm^–1^, corresponding to –C=C―, and a broad –OH peak in the range between 3200 and 3700 cm^–1^. The spectra of cross-linker EGDMA exhibited the characteristic stretching vibrations of –C=C― at 1640 cm^–1^, corresponding to the presence of alkenes. The –C=C― band at 1640 cm^–1^ observed in the cross-linker (EGDMA) completely disappeared in the spectra of the cross-linked networks of the pHEMA hydrogels, as shown in Figure 5B. This disappearance indicates the consumption of double bonds during the cross-linking. In the spectrum of pure DMPA, the most intense bands were attributed to the vibrations of the methyl groups in the wide range of 2800–3000 cm^–1^, aromatic rings at 1577–1595 and 1455–1490 cm^–1^, carbonyl groups at 1690 cm^–1^, and C–O bonds at 1000–1160 cm^–1^, correlating with the results of Kaczmarek et al. (2010).^68^

**Figure 5:**
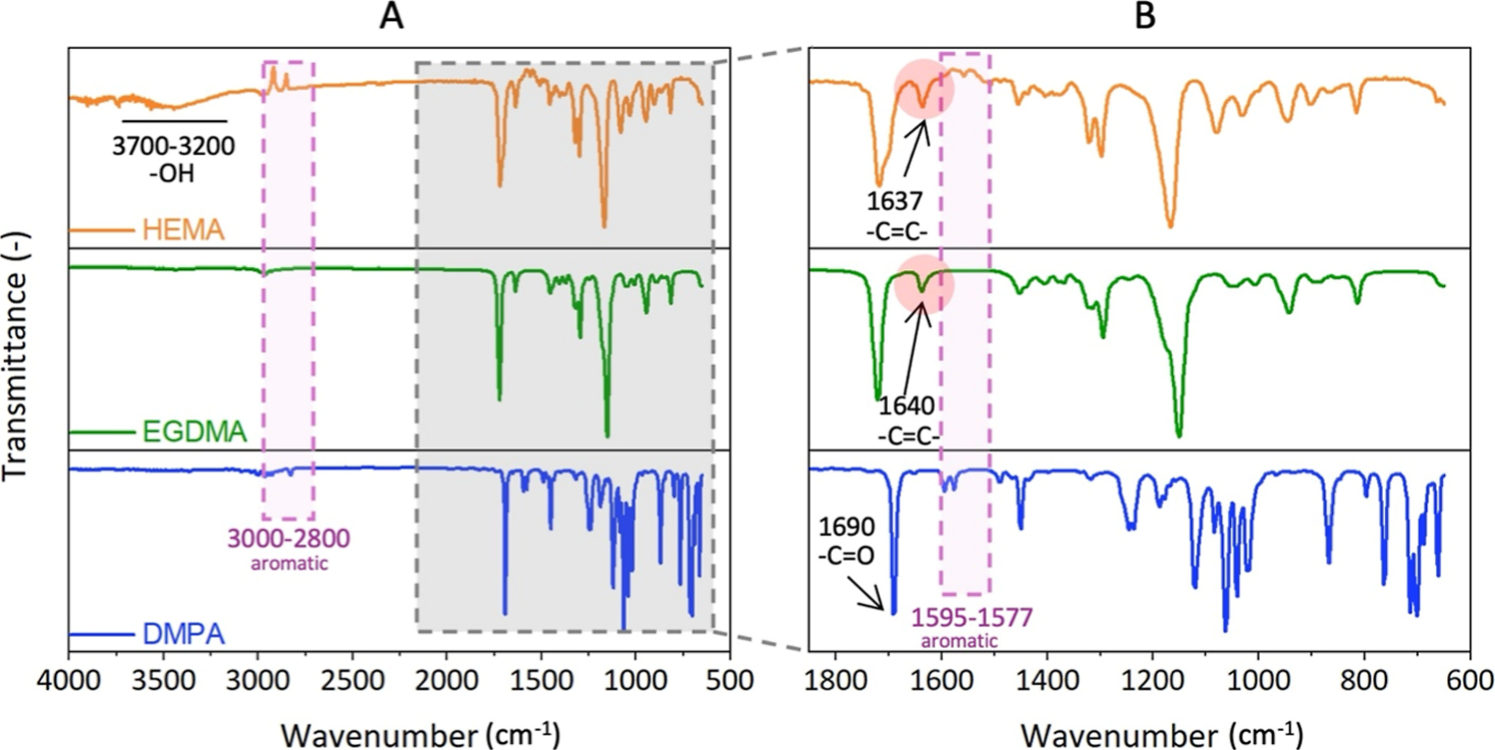
(A) Transmission ATR-FTIR spectra of the hydrogel precursors showing the spectra of the HEMA monomer, EDGMA cross-linker, and DMPA initiator; (B) enlarged profile of the marked region in (A). Marked in red are the alkene –C=C― peaks in HEMA and EGDMA disappeared during the formation of the cross-linked pHEMA network.

The chemical composition of the pHEMA hydrogel surface was evaluated in the dry state. The spectra of the pHEMA hydrogels prepared in both laboratory and nitrogen atmospheres are shown in Figure 6. In both spectra, the broad bands within the range of 3600–3150 cm^–1^ are attributed to the stretching vibrations of the hydroxyl groups (O–H).^69,70^ In the region of 1760–1650 cm^–1^, the bands at 1723 cm^–1^ are attributed to the stretching vibrations of the carbonyl groups (C=O).^71,72^ Additionally, in the region 1404–1379 cm^–1^, the bands correspond to the bending vibrations of methylene groups (―CH_2_―), and the bands in the region 1321–1032 cm^–1^ are attributed to the stretching vibrations of the C–O–C groups. The pHEMA hydrogel prepared in the laboratory atmosphere showed (at 1690 cm^–1^) the photoinitiator DMPA photolysis residues, such as methylbenzoate, 1,2-diphenylethane-1,2-dione or 2,3-diphenylbutane-2,3-diol,^73,74^ indicating the origin of the wrinkling patterns. In the nitrogen atmosphere, there is no termination of DMPA radicals by atmospheric oxygen. As a result, during the synthesis of *pHEMA N*_2_, a hydrogel with a smooth surface is formed unlike in the case of synthesis under laboratory conditions, yielding a wrinkling surface of *pHEMA air*.

**Figure 6:**
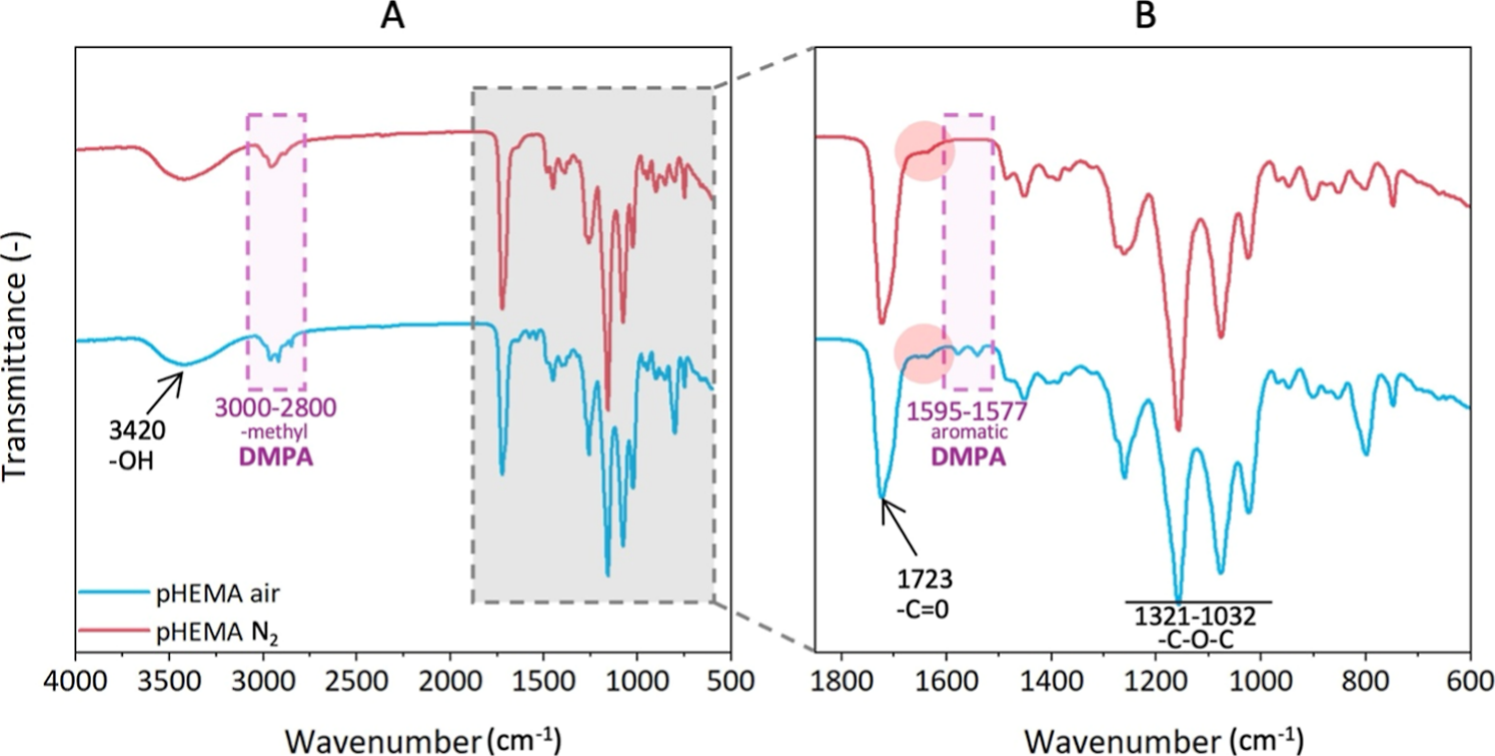
(A) Transmission ATR-FTIR spectra of the pHEMA hydrogels prepared in different atmospheres; hydrogels prepared under nitrogen atmosphere in red; and in standard laboratory atmosphere in blue. (B) Enlarged profile of the marked region in (A) showing the presence of photoinitiator residues in the spectra of *pHEMA air*.

### Mechanical Properties

The mechanical properties closely related to the flexibility of the pHEMA hydrogels were investigated immediately after their synthesis in the wet state using tensile tests. The engineering tensile stress–strain curves of *pHEMA air* and *pHEMA N*_2_ are plotted in Figure 7A, and a comparison of Young’s moduli and tensile elongation of the two hydrogels is shown in Figure 7B. The Young’s moduli were calculated from the linear-elastic region of the stress–strain curves, showing the *E*_t_ 0.92 ± 0.07 MPa and the tensile elongation of 101.96 ± 8.46% for the *pHEMA air* and 0.82 ± 0.05 MPa and 42.03 ± 5.06% for the *pHEMA N*_2_, respectively.

**Figure 7:**
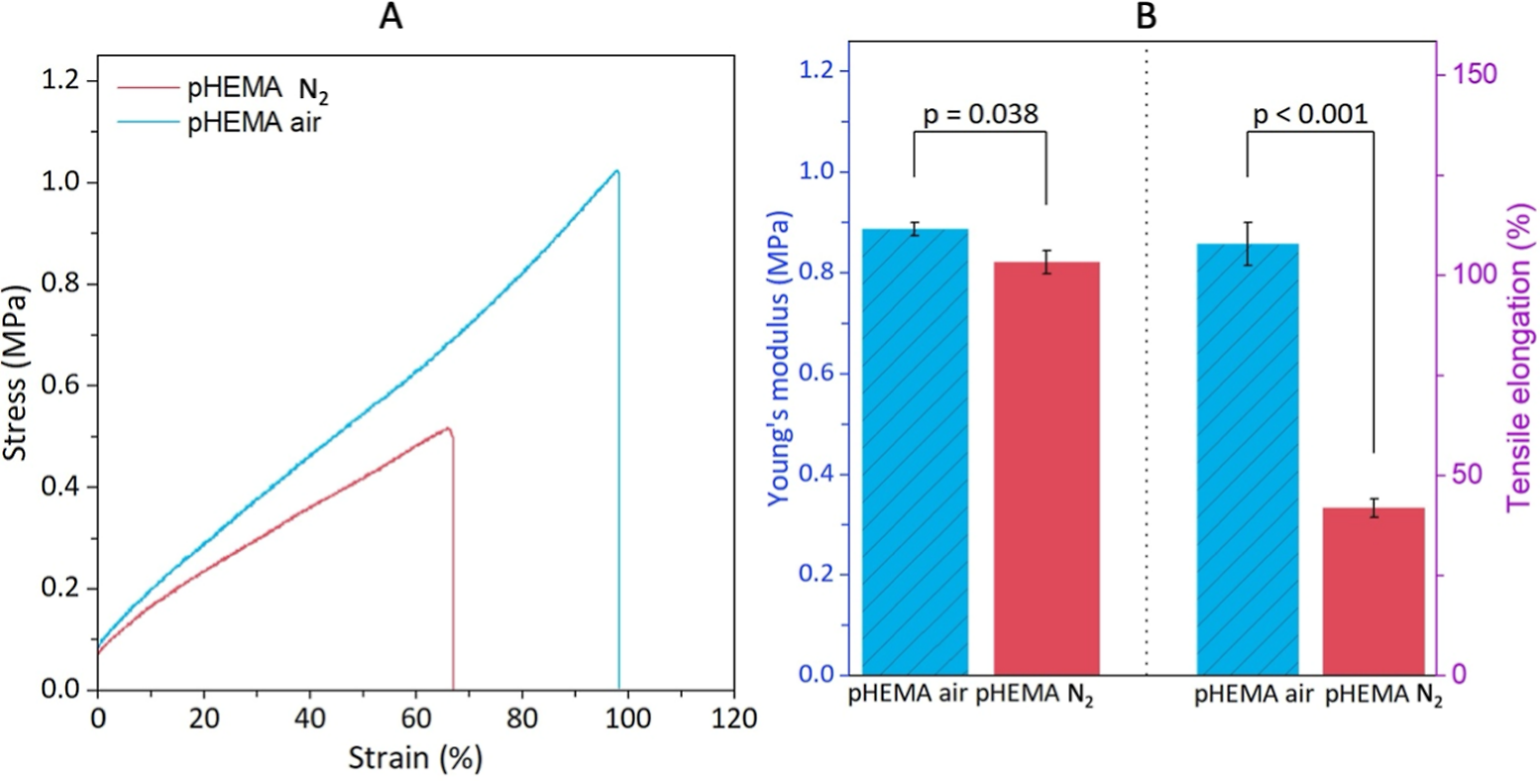
(A) Tensile curves of pHEMA hydrogels and (B) Young’s modulus (blue) and tensile elongation (pink) of pHEMA hydrogels.

Even though the results are comparable to the results of Alqahtani et al. (2023)^75^ and Zhu et al. (2013),^76^ most of the studies on the mechanical properties of pHEMA hydrogels are conducted in a wet or swollen state or in the form of thin films. In such studies, the Young’s modulus of 16.8 MPa has been achieved by Bose and Lau (2010)^77^ or between 0.25 and 59.4 MPa reported by Boazak et al. (2019)^78^ on pHEMA hydrogels containing EGDMA and vinyl methacrylate. Although the toughness of EGDMA-cross-linked hydrogels has been reported by Moghadam and Pioletti (2015),^79^ the tensile strength of dry HEMA-EGDMA-based materials has not yet been reported. However, the mechanical properties depend not only on the moisture content in the hydrogels but also on the testing conditions, including the parameters of preload and load speed.

To compare the hydrogel to the bovine AC, the reported elastic modulus for a healthy bovine cartilage is reported between 10.6 and 18.1 MPa under compressive loading at frequencies between 1 and 88 Hz.^80^ This makes bovine cartilage approximately twice as stiff as human cartilage. As a comparison, Petitjean et al. (2023)^81^ concluded that the elastic modulus ranged from 250 kPa to 3 MPa in compression, whereas Akizuki et al. (1986)^82^ reported a tensile modulus of AC between 1 and 15 MPa. To provide a comparison between the time-dependent behavior of the complex modulus for the pHEMA hydrogels and the cartilage samples, we performed dynamical mechanical analysis (DMA) to simulate the dynamic-compressive loading conditions typical of joint environments (Supporting Information C). Based on the results (Figure S3), both pHEMA samples showed lower moduli with a gradual increase over time, with the *pHEMA N*_2_ and *pHEMA air* reaching values of 93.2 ± 13.2 and 62.2 ± 8.9 kPa. In contrast, native cartilage exhibited a much higher modulus of 407.9 ± 43.3 kPa, comparable with the literature.^81^ Overall, the tensile strength of the prepared *pHEMA air* had properties that might be suitable for the cartilage laboratory model for the superlubricity experiments.

### Static Swelling Behavior

The swelling behaviors of two types of pHEMA hydrogels, *pHEMA N*_2_ with a smooth surface and *pHEMA air* with a wrinkled surface, were investigated using two different fluids: commercial PS and SF. These findings were compared with the swelling behavior of bovine AC in the same media. The data were fitted to first-order kinetic equations, and the first-order swelling constant *k*_1_ with the degree of swelling at equilibrium *S*_max_ was calculated, as shown in Table 1.

**Table 1:** Calculated First-Order Rate Constant *k*_1_, the Correlation Coefficient *R* ^2^, and the Predicted Degree of Swelling at Equilibrium *S*_max_ of the pHEMA Hydrogels and AC Samples in PS and SF^a^

	physiological solution			synovial fluid		
	pHEMA N_2_	pHEMA air	AC	pHEMA N_2_	pHEMA air	AC
*k*_1_ (h^–1^)	1.58 ± 0.40	1.38 ± 0.17	20.90 ± 13.49	1.29 ± 0.54	1.04 ± 0.29	27.57 ± 4.02
*R* ^2^ (−)	0.99 ± 0.01	0.98 ± 0.01	0.97 ± 0.03	0.98 ± 0.01	0.97 ± 0.02	0.93 ± 0.07
*S*_max_ (%)	49.97 ± 3.27	65.49 ± 8.51	164.37 ± 37.86	53.61 ± 2.40	60.32 ± 9.45	219.08 ± 54.01

a The calculated swelling ratios of the pHEMA and AC samples, fitted with the first-order kinetic model, are shown in Figure 8.

The swelling of the AC samples was significantly greater than that of both types of pHEMA hydrogels, both in PS and SF. The initial increase in fluid content was evident during the 30 min swelling period and the calculated *k*_1_, which in the case of PS was 20.90 ± 13.49 h^–1^ whereas in the case of SF reached 27.57 ± 4.02 h^–1^, both rate constants indicating a very fast initial swelling of the AC samples. At this point, the swelling ratio in PS reached 140.6 ± 39.6%, whereas that in SF reached 222.8 ± 43.3%. The AC samples were initially completely dried, and the cartilage matrix was rehydrated from its entire volume. The overall swelling capacity gradually decreased over time, until the equilibrium was reached at 202.4 ± 49.3% in the case of SF (predicted *S*_max_ 219.08 ± 54.01), compared to the 125.5 ± 35.8% of the AC samples submerged in PS (predicted *S*_max_ 164.37 ± 37.86), indicating a fluid-specific^83^ response of the AC to the SF containing hyaluronic acid, present in the human SF.

Owing to the substantial difference in osmotic pressure between the AC matrix and the surrounding fluid, the AC matrix rapidly absorbed the fluid and swelled. Moreover, a direct interaction between the collagen type II fibrils of the cartilage and the SF containing hyaluronic acid^84^ has affected the swelling, increasing the swelling capacity of the cartilage.^85^ Over time, the disparity in osmotic pressure decreased, the rate of fluid uptake diminished until equilibrium was reached, and net fluid movement into the cartilage stabilized. The osmotic properties of the surrounding fluid also explain why swelling is more pronounced in SF than in PS. The PS, which is a relatively “simple” saline solution (0.9% NaCl) containing mainly sodium and chloride ions with an osmotic pressure similar to that of extracellular fluid, exhibited a lower swelling capacity than SF.^77,78^ This is further supported by the greater osmotic pressure difference between the fluid and the AC matrix in SF.^86^ This finding supports the notion that natural cartilage has a superior capacity to absorb fluids, which is essential for proper joint function. Moreover, enhanced swelling in SF highlights the adaptability of bovine cartilage to the joint environment, where SF is naturally present.^87^ A paired comparison of the three materials is shown in Figure 9.

**Figure 8:**
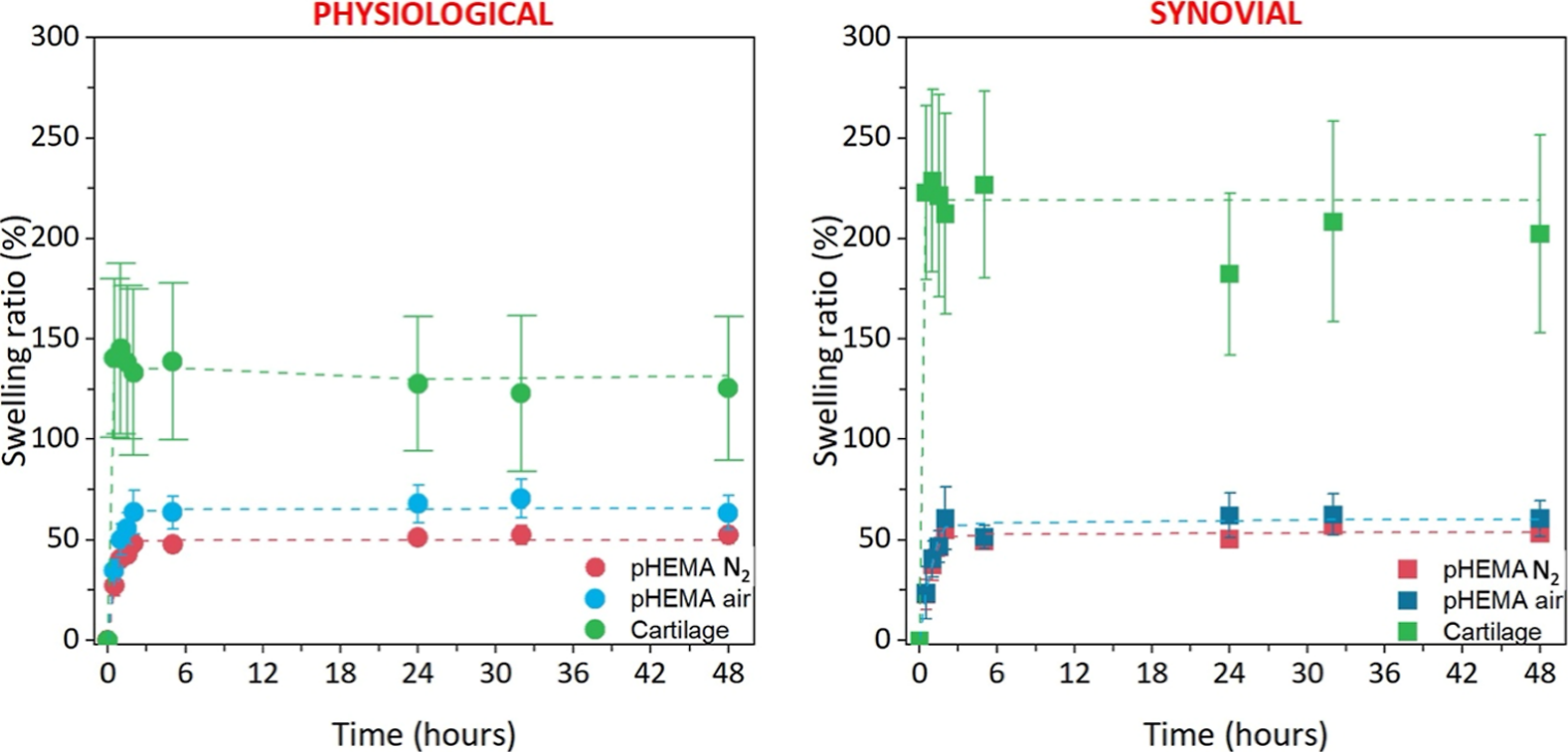
Swelling ratios of pHEMA hydrogels prepared under nitrogen and laboratory atmospheres compared with cartilage samples in physiological solution (left) and SF (right). The time points were fitted using a first-order swelling kinetic model.

**Figure 9:**
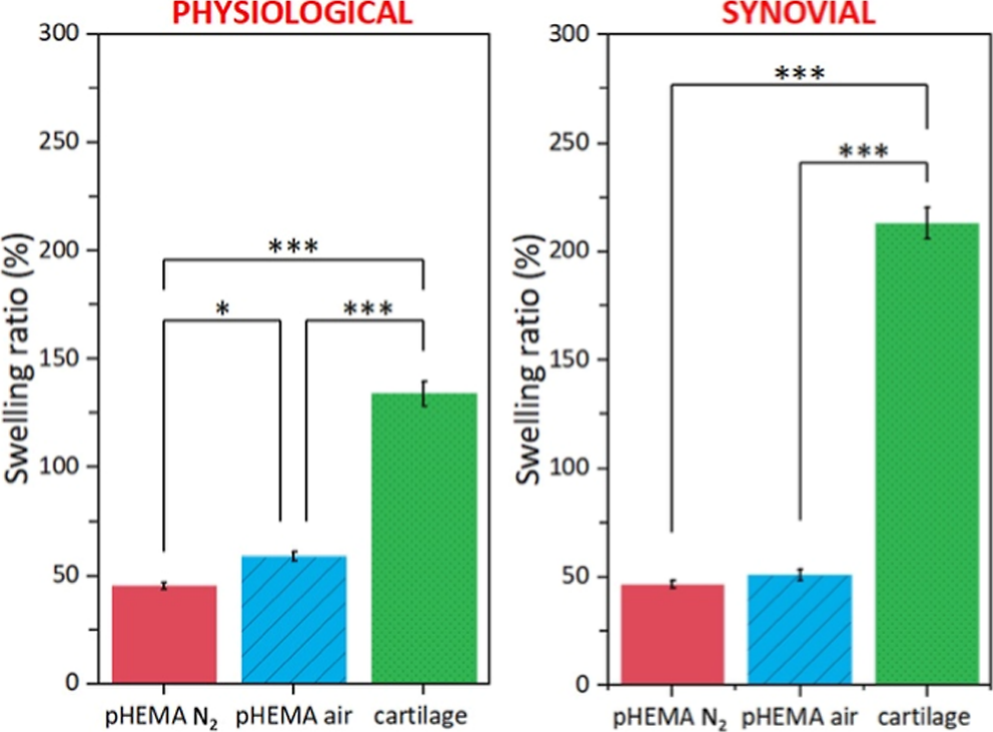
Paired comparison between swelling ratios (%) of the observed pHEMA hydrogels and AC samples in PS (left) and SF (right) at the end of the test. The results are presented as the mean ± SD of the mean, *n* = 5/group; *p*-values resulting from Tukey’s test with statistical significance (*p* < 0.05) are marked *, *p*-values reaching statistical significance (*p* < 0.01) are marked **, and *p*-values reaching significance (*p* < 0.001) are marked ***.

The osmotic effect of the swelling fluid was much less pronounced with the pHEMA hydrogels (mostly because of the regular structure of the hydrogel network compared with the collagen-based matrix of the cartilage). Looking at the swelling of the smooth *pHEMA N*_2_, the maximal swelling in the PS was 52.3 ± 4.1%, with a swelling rate of 1.58 ± 0.40 h^–1^, while in the SF, the swelling exhibited 53.5 ± 2.5% with the swelling rate of 1.29 ± 0.54 h^–1^, showing that the swelling rate and the maximal swelling capacity were similar in both media. Nevertheless, the results are comparable to those of swelling studies performed on similar pHEMA structures in a disodium phosphate buffer.^88^ On the other hand, the wrinkled *pHEMA air* exhibited maximal swelling in PS 48.3 ± 3.2% with a rate of 1.38 ± 0.17 h^–1^, while the hydrogels swelled up to 57.8 ± 3.8% with a rate of 1.04 ± 0.29 h^–1^ in the SF.

These results are comparable to our previous work on the pHEMA hydrogels with 60% w/w HEMA content (Krajňák et al., 2022),^51^ where the maximum swelling ratio was approximately 50% in Milli-Q water. In regard to the salt present in the PS, the pHEMA-based hydrogels show lower swelling with increasing content of the salt, as discussed by Baker et al. (1995)^89^ and Pan et al. (2020).^90^ Additionally, Refojo (1967)^91^ and Kim et al. (2004)^92^ showed that the solubility of hydrophobic pHEMA segments is decreased in the presence of electrolytes, strengthening the hydrophobic bonds and domains in the polymer.

In summary, despite the variation in the swelling of the cartilage and pHEMA hydrogels in the SF used in our tribological trials, the swelling kinetics of pHEMA hydrogels were remarkably consistent. A crucial consideration is that the swelling of *pHEMA air* is slightly greater than that of *pHEMA N*_2_ in the SF, implying that employing *pHEMA air* with a higher SF absorption capacity (compared to *pHEMA N*_2_) would be advantageous for future experiments, as we endeavored to achieve swelling behavior similar to that of AC.

### Tribology

The frictional behavior of *pHEMA N*_2_ and *pHEMA air* in contact with AC lubricated with artificial SF was investigated and the results are shown in Figure 10. To assess the ability of the pHEMA hydrogels to mimic AC behavior, the results were compared with measurements of cartilage-on-cartilage contact under the same conditions. In addition, a FT PVA hydrogel prepared using the methodology described in a study by Nečas et al. (2023)^52^ was included as a reference material, enabling a direct comparison between the two types of hydrogels.

**Figure 10:**
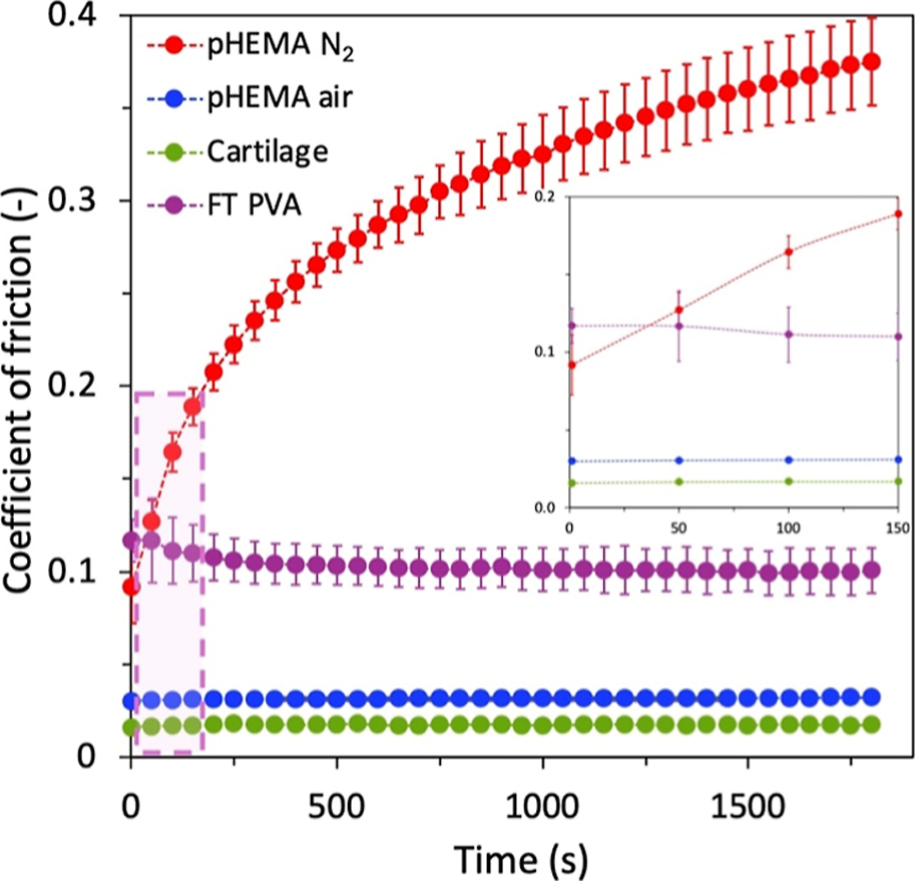
COF of pHEMA hydrogels prepared under nitrogen and laboratory atmospheres compared with FT PVA hydrogels and cartilage samples.

AC reported the lowest initial COF value of 0.0159 ± 0.0006, with only a slight increase during the 30 min frictional test. At the end of the measurements, the cartilage-on-cartilage contact exhibited a COF value of 0.0173 ± 0.0006. Comparatively similar results were obtained for *pHEMA air* with a wrinkled surface. The initial COF in the contact, 0.0299 ± 0.0030, as in the case of AC, increased only slightly to 0.0320 ± 0.0037. In contrast, *pHEMA N*_2_ with a smooth surface showed significantly different results. The initial COF value (0.0917 ± 0.0193) was many times higher than those of the other two materials and increased rapidly during the test. The final COF value of 0.3750 ± 0.0237 was an order of magnitude higher than those of AC and *pHEMA air*. The COF values of the FT PVA hydrogel were found to lie between those of the two types of pHEMA hydrogel. Although, the initial COF value of 0.1170 ± 0.0113 was even higher than that of *pHEMA N*_2_, the FT PVA hydrogel nearly maintained its initial COF, decreasing only slightly during the measurement to 0.1006 ± 0.0122.

When the AC or hydrogel contact is under load, it operates in a biphasic lubrication regime.^93^ The interstitial fluid is squeezed from the extracellular matrix into the contact area, and this exudation of the pressurized interstitial fluid helps to achieve low COF values. To sustain this effect over time, the contact bodies need to be adequately rehydrated, meaning that they must remain in a swollen state. In the cartilage-on-cartilage or cartilage-on-hydrogel configurations, rehydration can occur simply because of the migrating contact area.^94^ The results of *pHEMA N*_2_ evoke inadequate rehydration, which aligns with its lowest swelling ratio, low swelling rate in the SF, and the highest permeability (Table 2). Shi et al.^95^ reported a doubling of the COF values when the water content of the PVA hydrogel was reduced by approximately 15%. Preparation of pHEMA hydrogels in a laboratory setting and under an inert nitrogen atmosphere also resulted in different Young’s moduli (0.93 ± 0.07 MPa for *pHEMA air* vs 0.82 ± 0.05 MPa for *pHEMA N*_2_), which could influence COF values. Shi et al.^96^ also found lower COF values for PVA/PVP hydrogels as the compressive tangent modulus increased.

**Table 2:** Calculated Hydraulic Permeability Coefficients of *pHEMA N*_*2*_ and *pHEMA Air*

	permeability coefficient (m^4^/N s^–1^)	SD
*pHEMA N*_*2*_	2.17 × 10^–14^	2.81 × 10^–14^
*pHEMA air*	8.79 × 10^–15^	4.82 × 10^–15^
human AC	1 × 10^–15^ to 10^–16^	** ^102–110^

Furthermore, different environmental conditions during pHEMA hydrogel preparation led to variations in surface morphology (smooth vs wrinkled surface). According to Rudge et al.^97^ under a certain level of normal load, surface asperities are flattened, leading to a smoother surface where the COF is determined by the material properties. However, the deformation of surface asperities causes a local increase in contact pressure, resulting in greater pressurized fluid exudation from the hydrogel matrix to the contact area. Additionally, surface wrinkles create a larger surface area, from which the pressurized interstitial fluid can be squeezed into the contact area. A wrinkled surface could also promote the capture of proteins and SF molecules, thereby supporting the formation of a boundary lubricating layer on the hydrogel surface and reducing COF. Moreover, during the preparation of *pHEMA air* hydrogel, the presence of oxygen has been shown to inhibit radical polymerization near the surface.^98^ Consequently, a gradient cross-linking to a specific depth is formed during photopolymerization. This surface layer, characterized by a less regular polymer network due to a lower degree of cross-linking, is softer, more water-saturated, has higher permeability and very low COF.^99^ The low permeability of the overall *pHEMA air* volume maintains a high level of interstitial fluid pressurization, which helps to maintain a low friction. At the same time, the presence of a surface layer enables a faster fluid exchange and promotes the formation of a thin lubricating film. This functional permeability enables the lubrication layer to regenerate between cycles without significant fluid loss from the hydrogel volume.

The differences between pHEMA and PVA can arise from several factors. In a study by Yarimitsu et al. (2016),^100^ FT PVA hydrogel prepared using a very similar methodology reported a high water content up to 84.4%. In another study, Murakami et al. (2021)^101^ reported a permeability of 2.0 × 10^–13^ m^4^/N s^–1^ and a Young modulus of 110 kPa for FT PVA hydrogel. Therefore, the FT PVA hydrogel probably has the highest water content but also the highest permeability. So, if the hydrogel is loaded, the water is excessively squeezed out of the hydrogel, leading to increased friction. However, the friction of FT PVA was maintained below the values of *pHEMA N*_*2*_. This could be due to the hydrophilic nature of PVA. PVA has numerous hydroxyl groups along its polymer chain that can form hydrogen bonds with water molecules squeezed from the hydrogel structure. Nevertheless, a more in-depth analysis of the differences between pHEMA and PVA will be conducted in a subsequent study. It is also important to acknowledge that long-term durability, wear resistance, and fatigue behavior are critical to this application. In this regard, we have conducted additional experiments to assess the hydrogel’s performance under prolonged loading, including analysis of surface damage, roughening, and potential material loss using post-test morphological evaluation. Our preliminary findings suggest that under moderate testing conditions, the pHEMA hydrogels maintain surface integrity (Figures S1 and S2), however, further systematic evaluation of wear characteristics under prolonged loading is ongoing.

### Permeability Coefficient of pHEMA Hydrogels

The ability of cartilage to support loads through interstitial fluid pressurization is dependent on its low hydraulic permeability. Human AC permeability is influenced by factors, such as tissue depth, applied pressure, and water content. In this study, permeability was calculated based on the stress relaxation behavior following the method outlined by Yamaguchi et al. (2018).^59^ The permeability values of human cartilage typically range from 10^–15^ to 10^–16^ m^4^·N s^–1^,^102–110^ with a tendency to decrease with increasing depth and increase under higher pressures.^103,111^ These values are consistent with the results presented in Table 2 for *pHEMA air*. Additionally, *pHEMA N*_*2*_ exhibited a slightly higher permeability coefficient in comparison to that reported for human cartilage.

The relationship between cartilage permeability and friction is complex and multifaceted. Increased permeability, often associated with cartilage degradation, can lead to reduced interstitial fluid pressurization, resulting in higher friction coefficients.^108,112^ This fluid pressurization is vital for load support and maintaining low friction in the AC.^112,113^ The depletion of glycosaminoglycans (GAGs), which increase permeability, leads to higher friction owing to a reduction in biphasic lubrication.^114^ Conversely, reinforcing cartilage with an interpenetrating polymer network can lower the friction by enhancing the interstitial fluid phase.^115^ Additionally, increased leakage at the osteochondral junction, often observed in conditions such as OA, can accelerate fluid depressurization, further elevating friction levels.^116^ Hence, in terms of permeability, *pHEMA air* is relatively similar to healthy human cartilage, whereas *pHEMA N*_*2*_ is more comparable to osteoarthritic AC. However, the progression of OA is also connected with the degradation of the AC structure and an increase in surface roughness, which is contradictory to the smooth surface of *pHEMA N*_*2*_. Therefore, *pHEMA N*_*2*_ cannot serve as a trustworthy OA cartilage-like model.

## Conclusions

Our research has demonstrated that a relatively simple and very known polymeric material, such as a poly­(hydroxyethyl methacrylate) (pHEMA) hydrogel, can prove to be a suitable alternative to the commonly used poly­(vinyl alcohol) (PVA) hydrogels in tribological experiments. By creating a defined atmosphere, it is possible to prepare a wrinkled transparent material similar to the AC in terms of morphology, mechanical properties, and COF, thereby demonstrating the potential of pHEMA hydrogels as a suitable tribological model material. Moreover, the easily achieved transparency of these materials can be advantageous for future experiments utilizing fluorescence microscopy, where the material’s transparency is among the most important parameters for successful imaging.

The potential of this material for superlubricity can be further enhanced by exploring various lipid compositions and their interactions with the porous structure of the hydrogel. The ability to fine-tune the properties of pHEMA hydrogels through controlled atmospheric preparation opens possibilities for creating customized gradient layered models that mimic specific stages of cartilage health or disease. Furthermore, the superior tribological performance of pHEMA hydrogels compared to PVA could lead to more accurate and reliable results in future studies of joint mechanics and cartilage degeneration.

## Supplementary Material



## Data Availability

Supporting data associated with this article can be found online as Data set at Zenodo: 10.5281/zenodo.14824950.

## Notes

COF of *pHEMA air* against cartilage during prolonged wear tests of; morphology of *pHEMA air* before and after the prolonged wear tests; and DMA of *pHEMA air*, *pHEMA N*_*2*_, and *cartilage* (DOCX)

## Abbreviations

AC: articular cartilage
AFM: atomic force microscopy
ANOVA: analysis of variance
ATR-FTIR: attenuated total reflectance Fourier transform infrared spectroscopy
CD: cast-drying
COF: coefficient of friction
DC: diffusion coefficient
DMPA: dimethoxy-2-phenylacetophenone
ECM: extracellular matrix
EGDMA: ethylene glycol dimethyl acrylate
FT: freeze–thaw
FTIR: Fourier transform infrared spectroscopy
GAGs: glycosaminoglycans
HA: hyaluronic acid
HEMA: 2-hydroxyethyl methacrylate
OA: osteoarthritis
PAA: poly­(acrylic acid)
PAM: poly­(acrylamide)
PANa: sodium phytate
PBO: poly­(*p*-phenylene-2,6-benzobisoxazole)
PBS: phosphate buffer
PE: poly­(ethylene)
PEG: poly­(ethylene glycol)
PGF: phosphate glass fibers
pHEMA: poly­(hydroxymethyl methacrylate)
PVA: poly­(vinyl alcohol)
PS: physiological solution
SD: standard deviation
SF: synovial fluid
