# Supplementary material for: Biomimetic pHEMA Hydrogels as an Alternative Cartilage-like Model Material for Biotribological Evaluations
Source: ACS Omega. 2025 Sep 15;10(38):44147–61. doi: 10.1021/acsomega.5c05569 (PMC12489733; doi:10.1021/acsomega.5c05569)
Supplement: Supplementary file 1 [file ao5c05569_si_001.docx]

**Supplement for the article ‘Biomimetic pHEMA hydrogels as an alternative cartilage-like model material for biotribological evaluations’**

Zuzana Kadlecova^1^, Ivana Chamradova^1^, Klara Tuslova^1^, David Rebenda^2,3^, Pavel Cipek^2^, Jan Gregora^2^, Alexandra Stredanska^2^, Yoshinori Sawae^4^, Premysl Mencik^5^, Martin Vrbka^2^, Lucy Vojtova^1,*^

^1^ Advanced Biomaterials Group, Central European Institute of Technology, Brno University of Technology, 621 00 Brno, Czech Republic ^2^ Biotribology Research Group, Faculty of Mechanical Engineering, Brno University of Technology, 616 69 Brno, Czech Republic
^3^ Centre of Polymer Systems, University Institute, Tomas Bata University in Zlin, 760 01 Zlin, Czech Republic

^4^ Department of Mechanical Engineering, Faculty of Engineering, Kyushu University 744 Motooka, Nishi-ku, Fukuoka 819-0395, Japan

^5^ Institute of Materials Chemistry, Faculty of Chemistry, Brno University of Technology, 612 00 Brno, Czech Republic

Table of Contents

[A. Data availability 2](#_Toc206775257)

[B. Wear tests pHEMA – cartilage 2](#_Toc206775258)

[C. Dynamical mechanical analysis 3](#_Toc206775259)

# Data availability

Supplementary data associated with this article can be found online as Dataset at Zenodo: <https://doi.org/10.5281/zenodo.14824950>.

# Wear tests pHEMA – cartilage

To understand the wear of the pHEMA hydrogels, we tested a combination of pHEMA air and bovine cartilage in a 5-hour test. The morphology of hydrogel samples was evaluated before and after the test according to the methodology described in the original manuscript.

**Figure S1.** Coefficient of friction of pHEMA air prepared under laboratory atmosphere against cartilage pin over 5 hours.


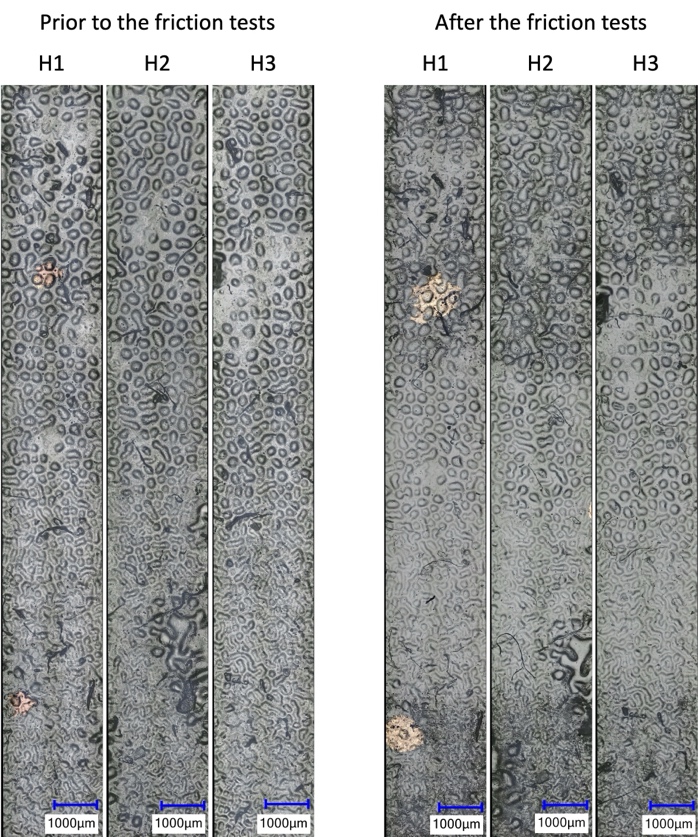


**Figure S2.** Morphology of pHEMA air prepared under laboratory atmosphere before (left) and after (right) the 5-hour wear test against cartilage pin.

# Dynamical mechanical analysis

Compression tests were performed on hydrogels swelled for 72h through dynamic mechanical analysis (DMA) using DHR2 (TA Instruments, USA) with a plate-plate geometry with 20 mm diameter. The measurements were performed in time sweep mode at constant temperature 25 °C, 1 Hz frequency, and 1% strain. For the compression tests, the samples were cut using a standardized punch with a 20 mm diameter.

The complex modulus was calculated from the measured storage and loss modulus for each type of sample. The data are represented as an average and standard deviation of (n = 4) measurements.


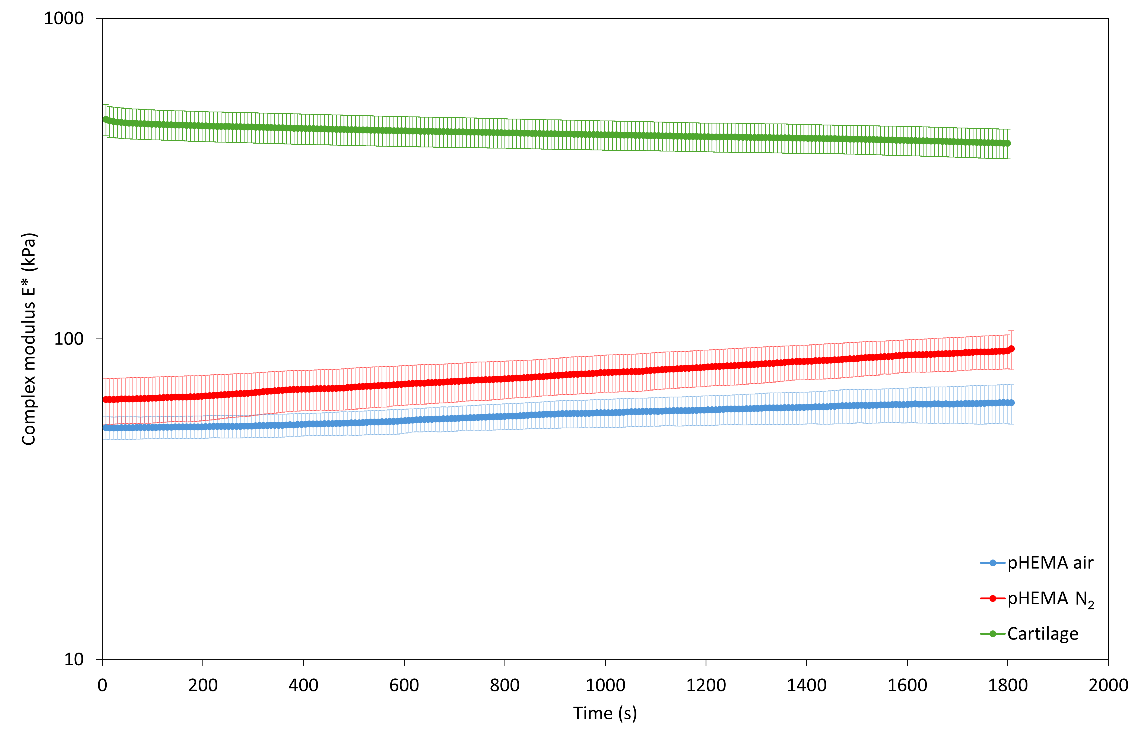


**Figure S3.** Complex modulus *E** of the pHEMA hydrogels synthesised under a nitrogen (*pHEMA N_2_*) and laboratory (*pHEMA air*) atmospheres compared to the complex modulus of the bovine cartilage.
